# Safety and efficacy of *Soyo-san* for the treatment of functional dyspepsia: A protocol for systematic review and meta-analysis

**DOI:** 10.1097/MD.0000000000022359

**Published:** 2020-09-25

**Authors:** Na-Yeon Ha, Ha-nul Lee, Hae-in Jeong, Seok-Jae Ko, Jae-Woo Park, Jinsung Kim

**Affiliations:** Department of Gastroenterology, College of Korean Medicine, Kyung Hee University, Seoul, Republic of Korea.

**Keywords:** functional dyspepsia, meta-analysis, protocol, *Soyo-san*, systematic review

## Abstract

**Background:**

Functional dyspepsia (FD) is a common condition characterized by gastrointestinal symptoms, such as abdominal fullness and epigastric pain. With the limitations of conventional Western medical treatments, symptoms often recur and lead to poor quality of life. *Soyo-san* (SYS) is a traditional herbal medicine that has been frequently used to treat indigestion. This protocol was designed to investigate the safety and efficacy of SYS for treating FD through a systematic review and meta-analysis.

**Methods:**

Trials will be searched from the following 11 electronic databases, up to March 2020: EMBASE, Medline (via PubMED), the Cochrane Central Register of Controlled Trials (CENTRAL), Allied and Complementary Medicine Database (AMED), Korean Medical Database (KMbase), KoreaMed, Korean Studies Information Service System (KISS), National Digital Science Library (NDSL), Oriental Medicine Advanced Searching Integrated System (OASIS), China National Knowledge Infrastructure Database (CNKI), and Citation Information by Nii (CiNii). Randomized controlled trials (RCTs) of SYS or modified SYS for FD will be included in this systematic review. The effects of control interventions such as placebo, no-treatment, and conventional Western medicine will be compared with those of SYS. RCTs investigating the synergetic effect of SYS and Western medicine compared with conventional Western medicine alone will also be evaluated. Two investigators will independently extract the data and assess the risk of bias in the included studies. The total clinical effective rate will be measured as the main outcome.

**Results:**

This systematic review will provide data on the use of SYS in the treatment of FD, based on indicators such as dyspepsia-related symptom score, recurrence rate, and adverse events.

**Conclusion:**

This study will determine the safety and efficacy of SYS for the treatment of FD.

**Review Registry Unique Identifying Number:**

reviewregistry969.

## 1 Introduction

Functional dyspepsia (FD) is a functional gastrointestinal disorder (FGID) characterized by the following symptoms: early satiation, postprandial fullness, epigastric pain, or epigastric burning.^[1]^ The prevalence of FD is between 5% and 11% globally.^[2]^ These symptoms frequently relapse and chronically affect patients, resulting in decreased quality of life and productivity, and increased economic burden.^[3,4]^ In FD patients, structural problems related to the symptoms are not identified on upper gastrointestinal examinations, such as endoscopy.^[5]^ It is known that the pathophysiology of FD may involve slow gastric emptying, insufficient gastric relaxation, and gastric hypersensitivity.^[6]^ Acid-suppression therapy, prokinetic agents, helicobacter pylori eradication therapy, and antidepressants are currently prescribed as standard treatment for FD. Nevertheless, due to the inefficacy of conventional medical therapies, up to 50% of FD patients resort to complementary and alternative medicine.^[7]^ As the pathophysiology of FD remains unclear and complex etiologies have been associated with various symptoms, a multi-target treatment like herbal medication may be useful.^[8]^

Soyo-san (SYS; Soyo-san in Korean, Xiaoyao-san in Chinese, or Shoyo-san in Japanese) is a traditional herbal formula that consists of the following crude herbs: Atractylodis Rhizoma Alba, Paeoniae Radix Alba, Poria, Bupleuri Radix, Angelicae Gigantis Radix, Liriopis Tuber, Glycyrrhizae Radix, Menthae Herba, and Zingiberis Rhizoma Recens.^[9]^ It has been prescribed for centuries to treat various conditions of the gastrointestinal tract, including abdominal pain, and abdominal distention after eating.^[10]^ Although a definite mechanism has not been established, it is known that some factors, including delayed gastric emptying, and visceral hypersensitivity, influence the manifestation of several FD symptoms. Among them, SYS is expected to relieve indigestion symptoms by acting on gastrointestinal motility disorders associated with emotional disorders such as depression and anxiety.^[11,12]^

A previous study systematically assessed the effects of SYS; however, the analysis compared SYS with only prokinetic medicines, and the outcomes could not be generalized.^[8]^ Thus, other conventional Western medicines and various parameters need to be reviewed to establish reliable evidence and formulate clinical practice guidelines for use in the treatment of FD. The purpose of this systematic review of randomized controlled trials (RCTs) is to confirm the safety and efficacy of SYS in the treatment of FD.

## 2 Methods

### 2.1 Inclusion criteria for study selection

#### 2.1.1 Types of studies

RCTs and quasi-RCTs will be included in this systematic review. Animal studies, cell experiments, case reports, non-RCTs, and commentaries will be excluded.

#### 2.1.2 Types of participants

The FD patients diagnosed with the Rome criteria will be included, and there are no region, sex, and age limitations. The Rome criteria are diagnostic criteria for the FGIDs. They were first announced in 1992 and last revised in 2016. For this reason, the studies conducted before 1991 will be selected after the agreement of 2 independent reviewers (HL and HJ), if their criteria are similar to the Rome criteria (e.g., no evidence of organic disease). Secondary symptoms, induced by conditions, such as gastric ulcer, gastroesophageal reflux disease (GERD), and irritable bowel syndrome (IBS), will be excluded. However, patients with GERD/IBS and FD diagnosed by Rome IV criteria will be exceptionally included, since Rome IV criteria demonstrate that FD may coexist with GERD or IBS.

#### 2.1.3 Types of interventions

RCTs of SYS will be included; SYS alone and modified SYS (herbs-added prescription). These interventions will be compared with SYS: placebo SYS, no treatment at all, and conventional Western drugs, including prokinetics, digestive enzymes, and antidepressants, among others. This systematic review will also compare the effects of combination therapy (SYS plus Western medicine) and Western medicine alone on FD.

#### 2.1.4 Types of outcome measures

The total clinical effective rate will be measured for the primary outcome. Secondary outcomes will include dyspepsia-related symptom score, functional dyspepsia symptoms rating, self-rating depression scale, self-rating anxiety scale, recurrence after treatment, and adverse events.

### 2.2 Search studies

#### 2.2.1 Database resources

We will search through the following 11 electronic databases from their beginning dates up to March 2020: EMBASE, Medline (via PubMED), the Cochrane Central Register of Controlled Trials (CENTRAL), Allied and Complementary Medicine Database (AMED), Korean Medical Database (KMbase), KoreaMed, Korean Studies Information Service System (KISS), National Digital Science Library (NDSL), Oriental Medicine Advanced Searching Integrated System (OASIS), China National Knowledge Infrastructure Database (CNKI) in Chinese, and Citation Information by Nii (CiNii) in Japanese. The fifth to ninth (v–ix) are Korean databases. The data from the Clinical Research Information Service and ClinicalTrials.gov will also be searched.

#### 2.2.2 Search strategy

The search terms, including symptom-related (e.g., indigestion, dyspepsia, disorder, discomfort, and stomach) and intervention-related (e.g., herbal medicine, *Soyo*, *Xiaoyao*, *Shoyo*), will be used independently or together in each database. For example, the specific search strategies for Medline (via PubMed) are presented in Table 1. Modified search strategies will be designed for other databases without language restrictions.

**Table 1:**
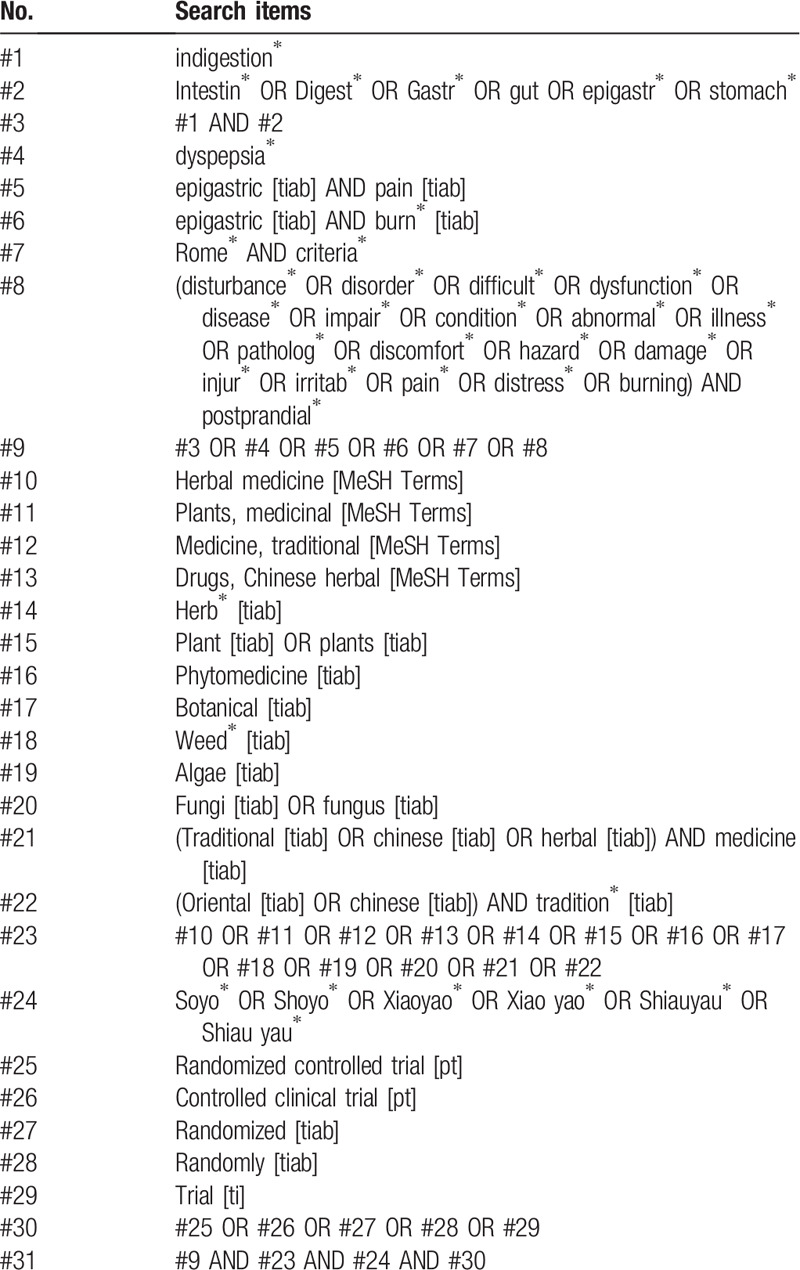
Search strategy used in PubMed.

### 2.3 Data collection and assessment

#### 2.3.1 Study selection

**Figure 1:**
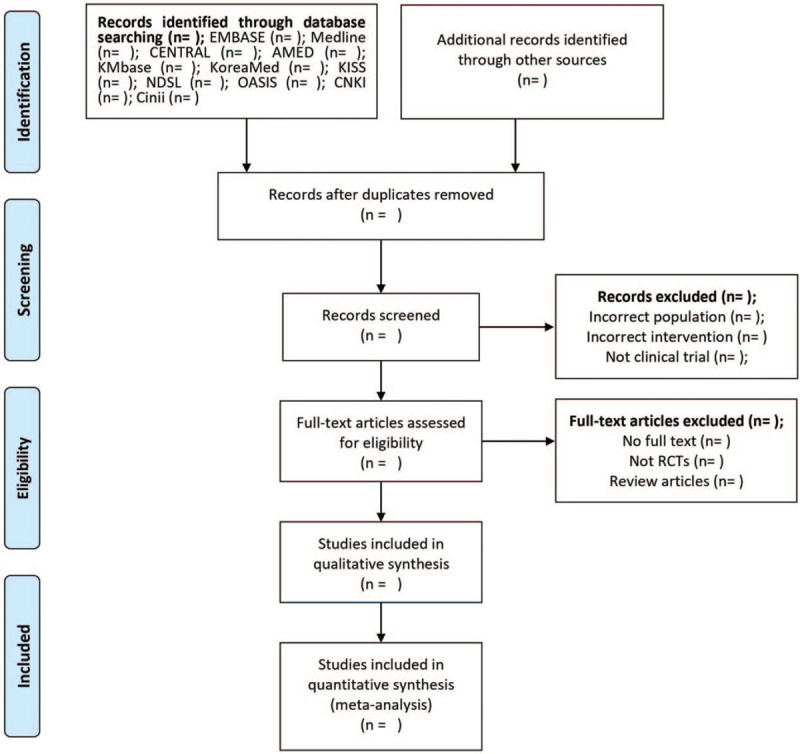
Flow chart of the search process. AMED = Allied and Complementary Medicine Database, CENTRAL = Cochrane Central Register of Controlled Trials, Cinii = Citation Information by Nii, CNKI = China National Knowledge Infrastructure Database, KISS = Korean Studies Information Service System, KMbase = Korean Medical Database, NDSL = National Digital Science Library, OASIS = Oriental Medicine Advanced Searching Integrated System, RCTs = randomized controlled trials.

After training on the selection procedure, 2 investigators (HL and HJ) will use Endnote X8 (Clarivate Analytics, Philadelphia) to upload the literature and independently review the titles and abstracts to exclude duplicate and irrelevant articles. During the screening, the independent reviewers (HL and HJ) will read the full-text articles and select studies that are appropriate for analysis. The details of the study selection process are summarized in the Preferred Reporting Items for Systematic Reviews and Meta-Analyses (PRISMA) flow diagram (Fig. 1). They will discuss all disagreements and reach a consensus unless the arbiter (NH) intervenes and makes adjustments.

#### 2.3.2 Data extraction

The standard form will be filled with the data, which would have been independently extracted by the 2 reviewers (HL and HJ). It will include the following information: the first author, year of publication, study design, interventions, treatment period, outcomes, and adverse events, among others. Any differences between the reviewers will be discussed and resolved by the arbiter (NH).

#### 2.3.3 Assessment of the risk of bias in included studies

Two reviewers (HL and HJ) will independently assess the risk of bias using the Cochrane collaboration's tool. It consists of the following items: random sequence generation, allocation concealment, blinding of participants and personnel, blinding of outcome assessment, incomplete outcome data, selective reporting, and other bias. Each result will be one of the following: low, unclear, and high. The reviewers will resolve all disagreements through discussion, and if necessary, the third researcher (NH) will intervene and settle the disagreement.

### 2.4 Data analysis

#### 2.4.1 Statistical analysis

The Review Manager program (version 5.3. Copenhagen: The Nordic Cochrane Centre, The Cochrane Collaboration, 2014) will be used for data synthesis. We will use the mean difference with 95% confidence interval (CI) for evaluating continuous data, and risk ratio or odds ratio with 95% CI for dichotomous data. A random-effects model will be included in the meta-analysis. The heterogeneity of included studies will be assessed by the *I*-squared statistic (*I*^*2*^ ≥ 50% means substantial heterogeneity) and the Chi-squared (*χ*^2^) test (*P* < .1 means statistical significance).

#### 2.4.2 Data synthesis

The efficacy of SYS will be synthesized and compared with the controls, including placebo, no-treatment, and conventional Western medicine. The effect of combined SYS and Western medicine will be compared with Western medicine alone to confirm the synergetic effect of combination therapy.

#### 2.4.3 Subgroup analysis

If there is high heterogeneity among selected studies, we will perform subgroup analysis. The cause of heterogeneity will be evaluated in terms of pattern identification in traditional Chinese medicine (TCM), species of added herbs and types of Western medicine, among others.

#### 2.4.4 Reporting bias analysis

If there are >10 articles for analysis, we will generate a funnel plot to assess publication bias.

#### 2.4.5 Sensitivity analysis

A sensitivity analysis will be conducted to confirm the robustness of the findings. The Consolidated Standards of Reporting Trials (CONSORT) and Extension for Herbal Interventions will be applied for assessment of methods and reporting quality of each trial.

#### 2.4.6 Grading the quality of evidence

The Grading of Recommendations Assessment, Development, and Evaluation (GRADE) will be used for assessing the quality of evidence.

#### 2.4.7 Handling missing data

We will try contact the authors of original research articles to obtain additional missing data (e.g., sending an e-mail). The intent-to-treat analysis will be used for assessing the data.

### 2.5 Ethics and dissemination

Identifying information of each participant will not be published, and this protocol does not need ethical approval. The systematic review will be published in a peer-reviewed journal and disseminated electronically.

## 3 Discussion

SYS is a well-known and widely used herbal medicine in Korea, China, and Japan. In a clinical study on 20 patients with FD using an electrogastrogram (EGG), *Jia*-*Wei*-*Xiao*-*Yao*-*San* decoction improved dyspeptic symptoms and normalized gastric motility.^[13]^ SYS has also been used to treat dyspeptic symptoms with psychological disorders such as depression. In TCM theories, dysfunction of the liver and spleen may cause abnormal digestion and can be cured by soothing liver-*qi* stagnation and promoting spleen transportation, interpreted as an effect of modulating neurotransmitter or hormone levels.^[10]^ In an animal study that used chronic multi-stressed rats, the administration of *Xiaoyao Powder* regulated serum corticosterone and gastrointestinal hormones.^[14]^ In a randomized, placebo-controlled trial of 180 perimenopausal women with FD and depression, *Xiaoyao pill* reduced depression, and improved the plasma levels of motilin and gastrin, and gastric emptying rate.^[10]^ A clinical study on FD patients with *Gan-qi* stagnation of *Pi-*deficiency syndrome type (FD-GP) showed that Modified Xiaoyao Powder alleviated clinical symptoms and regulated some parameters of EGG within normal range, elevating pharmacokinetic characteristics of ferulic acid, one of its active constituents.^[8,15]^ As mentioned above, SYS is often prescribed for FD patients with GP (“liver stagnation” and “spleen deficiency”), one of the syndromes of pattern identification in TCM. According to the TCM standard for diagnosing syndromes of FD, the “liver depression and spleen deficiency” type is characterized by symptoms such as stomach pain or discomfort, belching and acid reflux.^[10]^ Meanwhile, a meta-analysis of 14 RCTs of Modified Xiaoyao-san (MXS) for treatment of FD found that MXS, alone or combined with other prokinetic agents, was more effective than prokinetic drugs for treating FD. Although the high risk of bias makes the evidence weak, it is remarkable that a drug overdose can be managed with an alternative herbal medicine without causing any serious side-effects. Thus, more controlled trials must be conducted to evaluate the safety and efficacy of MXS in the treatment of FD.^[8]^

Although previous meta-analyses on the effects of SYS on FD have been performed, synergetic effects with only prokinetic drugs, as controls, were investigated and the evaluation of effectiveness was confined to symptom improvement.^[8]^ Most of the reviewed literature was Chinese studies, and the quality of the research methodologies was weak. In this systematic review of randomized controlled trials, we intend to review databases outside China to establish outcomes that can be generalized for clinical practice in various countries including Korea. In addition, control groups, including placebo and no-treatment groups, will be set and synergistic effects with drugs such as antidepressants, PPIs, and prokinetics will be analyzed. In the outcomes, detailed subjective symptoms and, if possible, effects on objective variables such as gastric emptying rate will be evaluated. Accordingly, it will be a more reliable study; it is designed to reflect the recent RCT trends on SYS, modified SYS, and SYS with Western medicine for the treatment of FD, and establish evidence for use in clinical practice.

Ethics approval is not required, because this systematic review is a literature-based study that will use already published data and no additional data will be collected. The results of the review will be published in a peer-reviewed journal and disseminated electronically or in print.

## Acknowledgments

This study was supported by a grant through the project “Development of Korean medicine clinical practice guidelines” of the Guideline Center for Korean Medicine, National Institute for Korean Medicine Development.

## Author contributions

**Conceptualization:** Na-Yeon Ha, Ha-nul Lee.

**Data curation:** Na-Yeon Ha, Ha-nul Lee, Hae-in Jeong, Seok-Jae Ko, Jae-Woo Park, Jinsung Kim.

**Formal analysis:** Jinsung Kim.

**Investigation:** Na-Yeon Ha, Ha-nul Lee, Hae-in Jeong.

**Methodology:** Seok-Jae Ko, Jae-Woo Park, Jinsung Kim.

**Resources:** Jinsung Kim.

**Writing – original draft:** Na-Yeon Ha.

**Writing – review & editing:** Jinsung Kim.
